# Gait analysis in clinically healthy sheep from three different age groups using a pressure-sensitive walkway

**DOI:** 10.1186/1746-6148-8-87

**Published:** 2012-06-22

**Authors:** Felipe S Agostinho, Sheila C Rahal, Fábio A P Araújo, Renato T Conceição, Carlos A Hussni, Alexander O El-Warrak, Frederico O B Monteiro

**Affiliations:** 1 Department of Veterinary Surgery and Anesthesiology, School of Veterinary Medicine and Animal Science, Univ Estadual Paulista (UNESP), Botucatu, SP, Brazil; 2 Department of Clinical Sciences, Faculty of Veterinary Medicine, University of Montreal, Montreal, Canada; 3 Universidade Federal Rural da Amazônia, Instituto de Saúde e Produção Animal, Belém do Pará, Brazil

## Abstract

**Background:**

Understanding normal gait requires allowing for variations in normal patterns by the sex, age, and species in question. Therefore, the aim of this study was to evaluate kinetic and temporospatial parameters in clinically healthy sheep from three different age groups with a pressure-sensing walkway. The sheep were judged to be healthy based on the results of complete physical and orthopaedic examinations and had no history of lameness. Twenty-one clinically healthy female Santa Ines sheep were divided into three groups: G1 – seven animals, aged from 8 to 12 months and weighing 19.5-33 kg; G2 - seven individuals, aged from 2 to 4 years and weighing 26.5-42 kg; and G3 - seven sheep, aged more than 5 years and weighing 37.3-45 kg. The animals were examined from two directions: first on the left side and then on the right side of the handler. The data from the first five valid trials in each direction were collected for each sheep and analysed using the designated software. A trial was considered valid if the sheep walked within the correct velocity (1.1-1.3 m/s) and acceleration (from −0.15 to 0.15 m/s^2^) ranges. The peak vertical force (PVF), vertical impulse (VI), gait cycle time, stance time, swing time, stride length, and the percentage body weight distribution among the four limbs were determined.

**Results:**

No significant differences were observed, in either the forelimbs or the hind limbs, between the left and right sides or between the two directions for any of the variables. No significant temporospatial differences were found among the groups. Significant PVF (%BW) differences were observed in the forelimbs (G1 > G3) and hind limbs (G1 > G3), and significant VI differences were observed in the forelimbs (G1 > G3).

**Conclusions:**

Young healthy sheep differ from older sheep in the vertical forces they create when walking at the same velocity on a pressure-sensing walkway.

## Background

Gait analysis is usually used to directly aid patient treatment and to better understand locomotion [1-3]. Several methods of gait analysis are available, including visual analysis, which depends on the ability and perspicacity of the investigator, and specific analysis, which requires specialised equipment [2-4].

Force platforms and pressure distribution sensors, such as pressure-sensing walkways, may be used to measure forces, moments, and accelerations [2,3]. Force platforms measure the orthogonal ground-reaction forces (vertical, mediolateral and craniocaudal) that result from limb positioning during locomotion, whereas pressure-sensing walkways measure vertical ground-reaction forces only [1-7]. Vertical forces have been commonly studied in animals due to their low variability and their magnitude relative to other orthogonal forces [1,4,6,8,9]. Craniocaudal and mediolateral forces may be affected by external factors and have smaller magnitudes [1,9].

Different pressure-measuring devices have been used to perform gait analyses in animals [7,10-14]. Some validation studies using dogs and horses have shown that certain kinetic measurements may differ when using a force platform rather than a pressure-sensing walkway [7,10,11], and highly accurate absolute force values may not be obtainable using a pressure plate [12]. However, several pressure-sensing walkway systems have been proven to be useful in clinical settings and for repeated gait assessments [11-13].

Additionally, some authors have suggested that experiments using pressure-sensing walkways are less time consuming, as walkways capture multiple sequential steps and as the contralateral limbs may be evaluated in the same trial [7,10,11,13,15].

Understanding normal gait requires understanding that normal patterns may vary by sex, age [2], and the species in question. Sheep have been used as an *in vivo* experimental model for orthopaedic research studies due to their size, availability and ease of handling, and they can sometimes provide an alternative to dogs [16-18]; some aspects of gait analysis of the sheep require more comprehension, however.

For example, a previous study using a pressure-sensing walkway reported that female Suffolk-mix sheep (with body masses ranging from 69.3 to 103 kg) are not suitable for gait analysis assessments, largely due to their flight zone and flocking behaviour [19]. In another study, however, female Merino-mix sheep (mean body mass 63 kg, range 46–90 kg) were trained to walk over a pressure-sensitive platform. The platform was used for nine weekly evaluations of a healing tibial defect [20].

Therefore, the aim of this study was to use a pressure-sensing walkway to evaluate kinetic and temporospatial parameters in clinically healthy female sheep from three different age groups. The hypothesis was that the older sheep would have altered kinetic parameters due to aging.

## Methods

This study followed the guidelines for the care and use of laboratory animals and was approved by the Institutional Ethics Committee.

Twenty-one clinically healthy intact female Santa Ines sheep were divided into three groups: G1 – seven animals, aged from 8 to 12 months and weighing 19.5-33 kg (mean ± SD, 25.78 ± 4.75 kg); G2 - seven individuals, aged from 2 to 4 years and weighing 26.5-42 kg (mean ± SD, 31.52 ± 4.88 kg); and G3 – seven animals, aged more than 5 years and weighing 37.3-45 kg (mean ± SD, 42.21 ± 3.11 kg).

The sheep were judged to be healthy based on complete physical and orthopaedic examinations and had no history of lameness. Before the data collection, the sheep were trained in being led by a halter twice each day for a period of approximately three weeks. Subsequently, the sheep were trained to walk across the walkway twice each day for a period of one week. In addition, hoof trimming was performed one week before the exams. Food was used to motivate the sheep to walk in all of the training sessions. Each sheep was weighed on a single electronic scale immediately before the data collection. All of the sheep were handled by a single handler.

### Data collection

The kinetic and temporospatial gait parameters were measured using a 1.951 mm x 447 mm pressure-sensitive walkway (Walkway High Resolution HRV4; Tekscan, South Boston, Massachusetts, USA) containing 33,408 pressure-sensing cells. The sensors of the pressure-sensing walkway were equilibrated and calibrated using a phantom according to the manufacturer’s specifications. During each trial, the sheep were led in a straight line over the pressure-sensing walkway by a handler using a halter. The animals were handled from two different directions: first on the left side of the handler and then on the right side. Food was used to motivate the animals.

Forty trials (20 in each direction) were captured for each animal. The data from the first five valid trials in each direction were collected for each sheep and analysed using the designated software (Walkway 7.0; Tekscan). A trial was considered valid if the sheep walked within the correct velocity (1.1-1.3 m/s) and acceleration (from −0.15 to 0.15 m/s^2^) ranges without head movement or pulling on the halter and if all four limbs had contact with the surface of the walkway during each walk cycle.

The temporospatial gait cycle time (s), stance time (s) swing time (s) and stride length (m) parameters were determined for each limb. The stance time percentage was calculated as follows: (stance time/gait cycle time) x 100. The swing time percentage was calculated as (swing time/gait cycle time) x 100. The stride corresponded to the distance between two consecutive ground contacts of the same limb.

The peak vertical force (PVF) and vertical impulse (VI) kinetic parameters were also determined. Both were normalised to the sheep’s body weight and represented as percentages of body weight (%BW and %BW*s). The percentage body weight distribution among the four limbs was calculated by (PVF of the limb/total PVF of the four limbs) x 100.

### Limb lengths and relative velocity

The limb lengths of the sheep were measure using retroreflective spherical markers (1.8 cm in diameter) that were tagged using a 3-camera kinematic system (Vicon MX-3+; Vicon, Oxford Metrics Group, Oxford, UK). The markers were placed on the skin by a single investigator using quick-drying glue. The forelimb markers were placed on the point of the cranial angle of the scapula, acromium of the scapulohumeral joint, lateral epicondyle of the humerus, styloid ulnar process, and distal lateral aspect of the III and IV metacarpus. On the hind limbs, the markers were placed on the greater trochanter of the femur, femorotibial joint between the lateral epicondyle of the femur and the fibular head, lateral malleolus and distal lateral aspect of the III and IV metatarsus. The length of each limb was determined by the sum of the distances between each pair of markers on that limb.

The relative velocities for the forelimbs and hind limbs were determined by the “Froude number”, which was defined as v^2^/gh, where v is the velocity, g is the gravitational acceleration and h is the height [12,37].

### Statistical method

The data were analysed using a linear repeated measurements model. For the temporospatial parameters and kinetic data, the animal side and the direction on the pressure-sensing walkway were considered to be the intra-sheep factors, while the group served as the inter-sheep factor. For the limb lengths, the animal’s side was considered to be the intra-sheep factor, and the group was the inter-sheep factor. The inter-sheep factor for body mass was the group. The sequential Bonferroni adjustment procedure was applied to our contrasts. The values were expressed as the mean ± standard deviation**,** and the coefficients of variation (CV) were calculated. A one-way ANOVA followed by Tukey's test was performed to compare the relative velocities of the forelimbs and hind limbs. Differences were considered significant at p < 0.05.

Pearson's correlation coefficients (r) were used to evaluate the linear relationships between the limb lengths and the PVF. The correlations were deemed significant at the 5% probability level.

## Results

After the training procedure, the sheep were able to walk appropriately on the pressure-sensing walkway.

No significant differences were found between the kinetic and temporospatial parameters of the left and right forelimbs or the left and right hind limbs when the group and the direction were not considered. None of the kinetic or temporospatial parameters, in either the forelimbs and or the hind limbs, differed significantly between the left and right sides or between directions when the group and the animal side were not considered.

The temporospatial values did not differ significantly among the groups in either the forelimbs or hind limbs (Tables 1 and 2). The forelimbs had significant differences in the PVF (G1 > G3) and VI (G1 > G3) (Table 3), while the PVF differed significantly (G1 > G3) in the hind limbs (Table 4).

**Table 1:** Comparison of the temporospatial parameters of the forelimbs among three groups (G1, G2 and G3) of healthy sheep

	**G1**		**G2**		**G3**		**P value**		
	**Mean ± SD**	**CV**	**Mean ± SD**	**CV**	**Mean ± SD**	**CV**	**G1-G2**	**G1-G3**	**G2-G3**
Stance Time (sec)	0.409 ± 0.020	5.07	0.426 ± 0.038	8.91	0.444 ± 0.025	5.65	0.30	0.04*	0.26
Swing Time (sec)	0.287 ± 0.008	3.02	0.301 ± 0.021	7.21	0.307 ± 0.015	5.16	0.12	0.03	0.51
Stride Time (sec)	0.701 ± 0.034	4.91	0.718 ± 0.053	7.49	0.751 ± 0.040	5.36	0.48	0.04*	0.17
Stride Length (m)	0.805 ± 0.032	3.97	0.848 ± 0.052	6.15	0.872 ± 0.057	6.52	0.11	0.019*	0.38
% of Stance	58.40 ± 0.83	1.42	59.33 ± 1.77	3	59.09 ± 0.730	1.24	0.20	0.36	0.71
%of Swing	41.07 ± 1.18	5.22	42.04 ± 0.99	2.36	40.91 ± 0.418	1.02	0.06	0.76	0.03

*Not statistically significant after alpha error correction.

**Table 2:** Comparison of the temporospatial parameters of the hind limbs among three groups (G1, G2 and G3) of healthy sheep

	**G1**		**G2**		**G3**		**P value**		
	**Mean ± SD**	**CV**	**Mean ± SD**	**CV**	**Mean ± SD**	**CV**	**G1-G2**	**G1-G3**	**G2-G3**
Stance Time (sec)	0.430 ± 0.021	4.93	0.446 ± 0.040	9.14	0.466 ± 0.035	7.66	0.39	0.06	0.27
Swing Time (sec)	0.267 ± 0.008	3.04	0.285 ± 0.018	6.34	0.278 ± 0.015	5.45	0.03*	0.15	0.39
Stride Time (sec)	0.689 ± 0.033	4.78	0.727 ± 0.056	7.78	0.742 ± 0.040	5.5	0.14	0.04*	0.53
Stride Length (m)	0.810 ± 0.040	5.04	0.842 ± 0.051	6.79	0.857 ± 0.057	6.7	0.25	0.10	0.58
% of Stance	62.20 ± 1.62	2.61	61.32 ± 1.72	2.82	62.77 ± 2.04	3.26	0.37	0.61	0.17
%of Swing	38.89 ± 1.14	2.94	39.31 ± 1.27	3.23	37.48 ± 2.00	5.35	0.62	0.08	0.03*

*Not statistically significant after alpha error correction.

**Table 3:** Comparison of the kinetic data of the forelimbs among three groups (G1, G2 and G3) of healthy Sheep

	**G1**		**G2**		**G3**		**P value**		
	**Mean ± SD**	**CV**	**Mean ± SD**	**CV**	**Mean ± SD**	**CV**	**G1-G2**	**G1-G3**	**G2-G3**
Peak Vertical Force (%BW)	54.67 ± 8.40^**a**^	15.36	47.23 ± 4.06^**ab**^	8.59	40.15 ± 3.83^**b**^	9.53	0.09	**0.001**	0.12
Vertical Impulse (%BW*s)	16.39 ± 2.53^**a**^	15.43	14.53 ± 1.30^**ab**^	8,94	12.46 ± 1.96^**b**^	15.73	0.30	**0.005**	0.20
% of Body distribution	31.38 ± 1.57	5.00	31.04 ± 1.20	3.86	31.60 ± 1.93	6.10	1.00	1.00	1.00

Values followed by different letters along each row are significantly different.

**Table 4:** Comparison of the kinetic data of the hind limbs among three groups (G1, G2 and G3) of healthy Sheep

	**G1**		**G2**		**G3**		**P value**		
	**Mean ± SD**	**CV**	**Mean ± SD**	**CV**	**Mean ± SD**	**CV**	**G1-G2**	**G1-G3**	**G2-G3**
Peak Vertical Force (%BW)	33.14 ± 5.06^**a**^	15.26	29.08 ± 2.63^**ab**^	9.04	23.52 ± 3.35^**b**^	14.24	0.25	**0.001**	0.06
Vertical Impulse (%BW*s)	9.51 ± 1.96	20.6	8.92 ± 1.15	12.89	7.74 ± 1.89	24.41	1.00	0.22	0.67
% of Body distribution	18.92 ± 1.12	5.91	19.05 ± 1.22	6.40	18.39 ± 1.81	9.84	1.00	1.00	1.00

Values followed by different letters along each row are significantly different.

The inter-group lengths differed in both the forelimbs (G1 < G3) and the hind limbs (G1 < G2 and G3) (Table 5). The velocity and the stride frequency did not differ significantly among the groups (Table 6), which was in contrast to a significant difference in body mass (G1 < G2 < G3) (Table 7). The relative velocity differed in both the forelimbs and hind limbs (G1 > G3). The average Froude numbers were 0.27 (G1), 0.25 (G2) and 0.23 (G3) for the forelimbs and 0.25 (G1), 0.24 (G2), and 0.23 (G3) for the hind limbs.

**Table 5:** Comparison of the forelimb and hind limb lengths (m) among three groups (G1, G2 and G3) of healthy sheep

	**G1**		**G2**		**G3**		**P value**		
	**Mean ± SD**	**CV**	**Mean ± SD**	**CV**	**Mean ± SD**	**CV**	**G1-G2**	**G1-G3**	**G2-G3**
Forelimb length	0.514 ± 0.022^**a**^	4.31	0.586 ± 0.029^**ab**^	5.03	0.607 ± 0.041^**b**^	6.82	0.125	**0.008**	0.397
Hind limb length	0.545 ± 0.022^**a**^	4.10	0.595 ± 0.019^**b**^	1.46	0.628 ± 0.073^**b**^	5.13	**0.001**	**< 0.001**	0.479

Values followed by different letters along each row are significantly different

**Table 6:** Velocity (m/s) and Stride frequency (cycles/min) among three groups (G1, G2 and G3) of healthy sheep

**Limb**	**G1**		**G2**		**G3**		**P value**		
	**Mean ± SD**	**CV**	**Mean ± SD**	**CV**	**Mean ± SD**	**CV**	**G1-G2**	**G1-G3**	**G2-G3**
Velocity (m/s)	1.175 ± 0.035	3.03	1.169 ± 0.034	2.91	1.185 ± 0.032	2.73	0.07	0.44	0.27
Stride frequency (cycles/min)	84.57 ± 5.47	6.48	83.98 ± 5.58	6.65	82.68 ± 5.80	7.02	0.58	0.06	0.16

**Table 7:** Values of body mass among three groups (G1, G2 and G3) of healthy sheep

	**G1**		**G2**		**G3**		**P value**		
	**Mean ± SD**	**(Maximum/Minimum)**	**Mean ± SD**	**(Maximum/Minimum)**	**Mean ± SD**	**(Maximum/Minimum)**	**G1-G2**	**G1-G3**	**G2-G3**
Body mass (kg)	25.78 ± 4.75^**a**^	19.5 - 33.0	31.52 ± 4.88^**b**^	26.5 - 42.0	42.21 ± 3.11^**c**^	37.3 - 45.0	**0.02**	**< 0.001**	**< 0.001**

Values followed by different letters along each row are significantly different.

The duty factors for the complete study population ranged from 0.55 to 0.62, with a mean of 0.58 (SD 0.01), for the forelimbs, and from 0.58 to 0.66, with a mean of 0.61 (SD 0.02), for the hind limbs.

## Discussion

In our study, characteristics that are considered unfavourable for performing gait analysis in sheep, such as flight zone and flocking behaviour [19], were reduced by training, as has been previously reported [20], and with the same halter when inducing the animals to walk on a pressure-sensing walkway. Another important strategy was using food to motivate the sheep to traverse the pressure-sensing walkway in a straight line. Moreover, the body mass (mean 33.10 kg, range 19.5-45.0 kg, comparable to that of a large-sized dog) of the sheep used in our study facilitated handling.

Although sheep reach sexual maturity at approximately 7–12 months of age, depending on the breed, the closure of the physeal plates of the long bones may occur as late as 36 months [16]. Thus, it is important to evaluate sheep of different ages, as in the present study, when evaluating skeletal growth.

Velocity measurements obtained by photoelectric cells and measurements from pressure-sensing walkway have been shown to be similar [7]. For this reason, only the latter measurement method was used. The designated software calculated the velocity by dividing the distance between consecutive foot strikes by the time between them [7,10,21]. The velocity used, 1.1-1.3 m/s, was considered comfortable for the sheep. The mean velocity used in a study assessing healthy sheep walking on a pressure-sensing walkway was 1.06 m/s [19]. In healthy dogs, the walking velocity on the same or similar type of pressure-sensing walkway has been reported to range from 0.5 to 1.14 m/s, depending on body sizes [10,21-24]. For healthy cats, the mean velocity has been reported to range from 0.6 to 0.81 m/s [25-27].

The duty factor in this study was >0.5, with a mean of 0.58 for the forelimbs and a mean of 0.61 for the hind limbs. In a study of healthy Suffolk-mix sheep, the duty factors were 0.66 and 0.69 for the forelimbs and hind limbs, respectively [19]. As has been reported previously [19], these values suggest a walking speed, as a duty factor >0.5 in the hind limbs is indicative of walking and a factor <0.5 is indicative of trotting or running [28].

Velocity and acceleration must be controlled because of their influence over the stance time, which is associated with the VI [2,10,29]. Studies of dogs have reported that velocity variations of 0.3 m/s may modify the ground reaction forces; as the velocity increased, the PVF increased and the VI decreased [4,30]. In the present study, the velocity was similar among the groups and had a mean value of 1.17 m/s (SD 0.02). In another sheep study, the mean velocity was 1.06 m/s, but the velocity varied from 0.57 to 1.49 m/s in the forelimbs and from 0.57 to 1.76 m/s in the hind limbs [19].

The two directions in which the sheep walked on the pressure-sensing walkway did not show significant differences, suggesting that the side of the handler did not interfere with the values. The direction of locomotion relative to the camera did not influence any of the temporospatial or kinetic parameters evaluated in a prior sheep study [19].

In humans, the gait of children is different from that of adults. Characteristics such as the cycle time, stride length and velocity change with growth [2]. In this study, no differences were detected in the temporospatial parameters among the groups, but the sheep moved at the same velocity. Stride length is diminished in healthy elderly humans [2,31]. This finding was not observed in Group 3, probably because the animals were younger adults. Sheep life expectancy has been reported to be approximately 10 to 12 years [32].

In addition, a relationship between stride length and height has been found in humans [2]. No stride length differences were found among the groups in our study, although the G1 animals had shorter limbs than those in other groups. The 8.2 cm and 6.7 cm disparities in the forelimbs and hind limbs, respectively, may not have been sufficient to cause detectable temporospatial differences. Additionally, a kinematic study of horses found that the duration of stance and stride in foals can be normalised using linear or dynamic scaling by the height at the withers and used to predict the values observed in adulthood. However, the height at the withers of the foals had increased by 30 cm when they reached adulthood [33].

In general, the stance and swing phases account for approximately 60% and 40%, respectively, of the gait cycle in healthy humans [2]. In the present study, the distribution was 58.86% stance phase and 41.49% swing phase for the forelimbs versus 61.88% stance phase and 38.85% swing phase for the hind limbs. Another study of sheep reported a mean of 66.31% for the forelimbs and 68.89% for the hind limbs in stance phase and 33.69% for the forelimbs and 31.11% for the hind limbs in swing phase [19].

The walking PVI (%BW) is generally higher for the forelimbs than the hind limbs when measured using a pressure-sensitive walkway [10,19,22,26,34]. Although this finding is consistent with the results from all of the groups in our study (mean values of 47.35% for the forelimbs and 28.58% for hind limbs), the values showed some variations compared to previous studies of healthy sheep (means of 52.52% and 38.52% for the forelimbs and hind limbs, respectively) [19], healthy dogs (means of 58.11% and 42.30% for the forelimbs and hind limbs, respectively) [10], and healthy cats (means of 48.2% and 38.3% for the forelimbs and hind limbs, respectively) [26].

The differences in velocity and calibration [26] and the animal species in question may have contributed to these differences in reported values. Differences in the calibration protocol may influence the number of activated sensors and result in differing readings [14,26,35]. In the present study, the calibration was performed according to the manufacturer’s guidelines using a phantom (a short three-legged stool). Weight was added to the stool to match the weight of each animal, and hard rubber was attached to the bottom of the feet of the stool to mimic a sheep’s hoof. Few studies have specified which methods were used to calibrate their pressure-sensitive walkways [7,11,14,26], which may explain the different measurements. In a study that evaluated vertical limb forces in dogs at a trotting velocity, for example, a 65 kg human subject placed one foot on the mat for calibration [7]. In a kinetic evaluation of cats, the mat was calibrated by having a 50 kg human subject stand on it with both bare feet [26].

The body weight distribution among the limbs of healthy sheep and dogs during walking has been described as approximately 30% on each forelimb and 20% on each hind limb [19,36]. Similar values were observed in our study, with 31.34% of the body weight placed on each forelimb and 18.79% placed on each hind limb.

In a study of healthy dogs walking on a force plate, the peak vertical forces were inversely correlated with physical size [36]. A similar correlation was observed in the present study for the forelimbs (R = −0.57) and hind limbs (R = −0.44) Thus, the PFV values for the forelimbs and hind limbs were greater in Group 1 than in Group 3, with Group 3 being composed of larger sheep. Although body size of the Group 2 was similar to Group 3, no significant PFV differences were observed. Furthermore, the relative velocity was greater in Group 1 than in Group 3, which may have contributed to the differences in the PVF values.

However, the VI of the forelimbs was higher in the G1 animals than in the G2 animals. The velocity may influence the stance time and consequently the VI [29], as the impulse reflects the association between force and the time that the foot is on the ground [4]. As a constant velocity was maintained in our study and as the stance time was statistically indistinguishable among the groups, the differences were associated with the different forces**.** These results are in contrast with those from another canine study in which the VI increased as the size of the dog increased [36], probably due to differences in velocity and individual dog size.

## Conclusions

The vertical forces created by young healthy sheep walking on a pressure-sensing walkway at the same velocity differ from those of older sheep under similar circumstances. This finding may be a source of variation and should be controlled in locomotion research studies.

## Authors’ contributions

FSA and SCR conceived and designed the study; FAPA and RTC helped collected the data; CAH and AOE assisted in data collection methods and FOBM helped draft the manuscript; all authors read, contributed to and approved the final manuscript.
